# Acute kidney injury in critically ill COVID-19 patients in a tertiary hospital: short and long-term kidney and patient outcomes

**DOI:** 10.1590/2175-8239-JBN-2024-0107en

**Published:** 2025-01-10

**Authors:** Juliana Alves Manhães de Andrade, Gisele Meinerz, Raphael Palma, Eduardo Rech, Marco Antônio Vinciprova Dall’Agnese, Cristiane Bundchen, Fernanda Bordignon Nunes, Gisele Branchini, Elizete Keitel

**Affiliations:** 1 Santa Casa de Porto Alegre, Porto Alegre, RS, Brazil.; 2 Universidade Federal de Ciências da Saúde de Porto Alegre, Programa de Pós-Graduação em Patologia, Porto Alegre, RS, Brazil.; 3 Universidade Federal de Ciências da Saúde de Porto Alegre, Porto Alegre, RS, Brazil.

**Keywords:** COVID-19, Acute Kidney Injury, Mortality

## Abstract

**Introduction:**

Acute kidney injury (AKI) in the setting of COVID-19 is associated with worse clinical and renal outcomes, with limited long-term data.

**Aim:**

To evaluate critically ill COVID-19 patients with AKI that required nephrologist consultation (NC-AKI) in a tertiary hospital.

**Methods:**

Prospective single-center cohort of critically ill COVID-19 adult patients with NC-AKI from May 1st, 2020, to April 30th, 2021. Kidney replacement therapy (KRT), recovery of kidney function, and death at 90-day and 1-year follow-up were evaluated.

**Results:**

360 patients were included, 60.6% were male, median age was 66.0 (57.0–72.0) years, 38.1% had diabetes, and 68.6% had hypertension. AKI stages 1, 2, and 3 were detected in 3.6%, 5.6%, and 90.8% of patients, respectively. KRT was indicated in 90% of patients. At the 90-day follow-up, 88.1% of patients died and 10.0% had recovered kidney function. Female gender (p = 0.047), older age (p = 0.047), AKI stage 3 (p = 0.005), requirement of KRT (p < 0.0001), mechanical ventilation (p < 0.0001), and superimposed bacterial infection (p < 0.0001) were significantly associated death within 90 days. At 1 year, mortality was 89.3%. Amongst surviving patients, 72% recovered kidney function, although with significantly lower eGFR compared to baseline (85.5 ± 23.6 vs. 65.9 ± 24.8 mL/min, p = 0.003).

**Conclusion:**

Critically ill COVID-19 patients with NC-AKI presented a high frequency of AKI stage 3 and KRT requirement, with a high 90-day mortality. Surviving patients had high rates of recovery of kidney function, with a lower eGFR at one-year follow-up compared to baseline.

## Introduction

Coronavirus disease-19 (COVID-19) became a health emergency a few months after the first case was described in Wuhan, China, in December 2019. Acute kidney injury (AKI) in the setting of COVID-19 infection is associated with worse clinical and renal outcomes^1,2,3,4^. Possible mechanisms for kidney damage include direct and indirect viral effects^5^, interacting with common and known risk factors for AKI^6^.

The demonstration of viral RNA^7,8,9^ and live virus^7^ in kidney biopsies supports the hypothesis of a direct tropism of SARS-CoV-2 to the proximal tubule cells and podocytes, possibly via angiotensin-converting enzyme-2 receptor expressed in these cells^10^. SARS-CoV-2 is a cytopathic virus that can directly induce renal tubular injury during infection and replication, and the immune response can promote fibrosis and apoptosis^5^. Acute tubular necrosis (ATN)^11,12^, thrombotic microangiopathy, and collapsing focal segmental glomerulosclerosis (FSGS) are reported histopathological findings^6^.

The indirect effects are those associated with critical illnesses, including hemodynamic instability, hypovolemia, hypoxia, cytokine storm, rhabdomyolysis, nephrotoxins exposure, endothelial dysfunction, coagulopathy, superimposed infections, and organ crosstalk^5,13^. Clinical and autopsy reports indicate that SARS-CoV-2 infection induces a hypercoagulation state that increases the risk of blot clots, including pulmonary, splenic, and renal infarction^6^.

The reported incidence of AKI in patients with COVID-19, especially those who are hospitalized, varies depending upon the severity of disease in the group of patients analyzed. In two large observational studies with over 5,000 patients hospitalized with COVID-19, AKI was reported in 32 to 37% of patients^14,15^. Approximately half of patients with AKI did not achieve complete recovery of kidney function by hospital discharge^14^. Independent predictors of AKI include age, race, gender, obesity, diabetes, hypertension, cardiovascular disease, low baseline estimated glomerular filtration rate (eGFR), higher interleukin-6 (IL-6) levels, or requirement of mechanical ventilation and vasoactive drugs^14,15^.

There are limited data on AKI progression and long-term outcomes in critically ill patients with COVID-19. Most reports evaluated in-hospital outcomes^13,14^ and up to 90-day^16,17^ outcomes, with fewer studies having longer follow-up^4,18^. As AKI is a clinically significant risk factor for chronic kidney disease (CKD), long-term sequelae of COVID-19-associated AKI warrants further investigation.

Our goal was to describe the clinical and laboratorial profile and subsequent clinical course of a sample of critically ill patients with COVID-19 and AKI that required a nephrologist consultation in a tertiary hospital. The main outcomes were 90-day mortality and recovery of kidney function, as well as survival and estimated glomerular filtration rate at one year.

## Methods

### Design

We conducted a prospective cohort study with adult patients admitted to the Intensive Care Units (ICUs) of a tertiary hospital in Southern Brazil due to COVID-19 infection between May 1st, 2020, and April 30th, 2021.

### Aim

Our primary goal was to evaluate short (90-day) and long-term (1-year) survival of a sample of critically ill patients with COVID-19 and AKI that required a nephrologist consultation (NC-AKI) in a tertiary hospital. The secondary goal was to describe the patients’ clinical and laboratorial profile, AKI stages, and need for kidney replacement therapy (KRT) during hospitalization. Lastly, the recovery of kidney function or dialysis need after hospital discharge and at one-year follow-up was evaluated.

### Patients

Adult (≥ 18 years old) patients with COVID-19 admitted to the ICU with NC-AKI. Patients with end-stage renal disease in chronic KRT were excluded.

### Ethics

The study was approved by the local ethics committee (protocol number: 4237991). An informed consent form was obtained from patients or their next of kin.

### Data Collection

Clinical and laboratory data were extracted from electronic medical records (EMR). Demographic data included age, gender, and body mass index (BMI). Comorbidities such as hypertension (HTN), obesity, diabetes mellitus (DM), malignancy, and coronary artery disease (CAD) were registered. Laboratory tests taken at admission and during follow-up included serum creatinine, C-reactive protein, D-dimer, ferritin, troponin-T, fibrinogen, lactate, and urinalysis.

### Definitions

COVID-19 infection was confirmed by quantitative real-time polymerase chain reaction (RT-PCR). Baseline creatinine level was defined as the mean creatinine value between 7 and 365 days before hospitalization retrieved from the EMR at the hospital or through the patient’s family. If that information was lacking, the minimum creatinine value during hospitalization was used as the baseline creatinine. AKI stages were defined according to KDIGO (Kidney Disease: Improving Global Outcomes) criteria as follow: stage 1 as an increase in serum creatinine of 1.5–1.9 times the baseline value or an increase in serum creatinine of 0.3 mg/dL within 48 hours; stage 2 as an increase of 2–2.9 times the baseline value; and stage 3 as an increase of at least 3 times the baseline value. Urine output was not used for the definition of AKI due to the scarce documentation of this variable. Positive albuminuria was defined as 2+ (or 1 g/dL) on urinalysis since lower values could be due to either ATN or acute interstitial nephritis (AIN). Frequency and modality of KRT – intermittent, continuous, or both – were reported. Peritoneal dialysis was not performed. Recovery of kidney function was independence of dialysis or serum creatinine within 50% of baseline according to Acute Dialysis Quality Initiative^19^.

### Outcomes and Follow-up

The primary outcomes within 90 days after COVID-19 infection included death, recovery of kidney function, and kidney function impairment with requirement of maintenance dialysis. Surviving patients had serum creatinine analysis at discharge and at 12 months. They were contacted by phone call and asked about their clinical status. Those who had missing values of serum creatinine were invited to collect new blood samples. Glomerular filtration rates were calculated using the CKD-EPI (Chronic Kidney Disease Epidemiology Collaboration) equation at baseline, discharge, and 12 months.

### Statistical Analysis

Continuous variables are presented as median with interquartile range (IQR) or mean and standard deviation (SD) according to their distribution. The normality assumption was verified by Shapiro-Wilk’s test. Categorical variables are expressed as absolute values along with percentages. For association between demographic data, clinical characteristics, and laboratory findings and death at 90 days and 1 year, χ^2^ test, Fisher’s exact test, and Mann-Whitney U test were used according to the nature and distribution of the variables. Variables with P < 0.20 were included in the multivariate analyses. Survival curves were constructed with Kaplan-Meier and compared by log-rank test. Cox proportional hazards regression analysis was used to determine hazard ratios at follow-ups. Creatinine values and eGFR at baseline, discharge, and one year after COVID-19 infection were compared with ANOVA for repeating variables and Sidak for multiple comparisons. The IBM SPSS^®^ v. 25 was used for data analysis. Statistic tests were two-sided, and levels of significance were 0.05.

## Results

**Figure 1:**
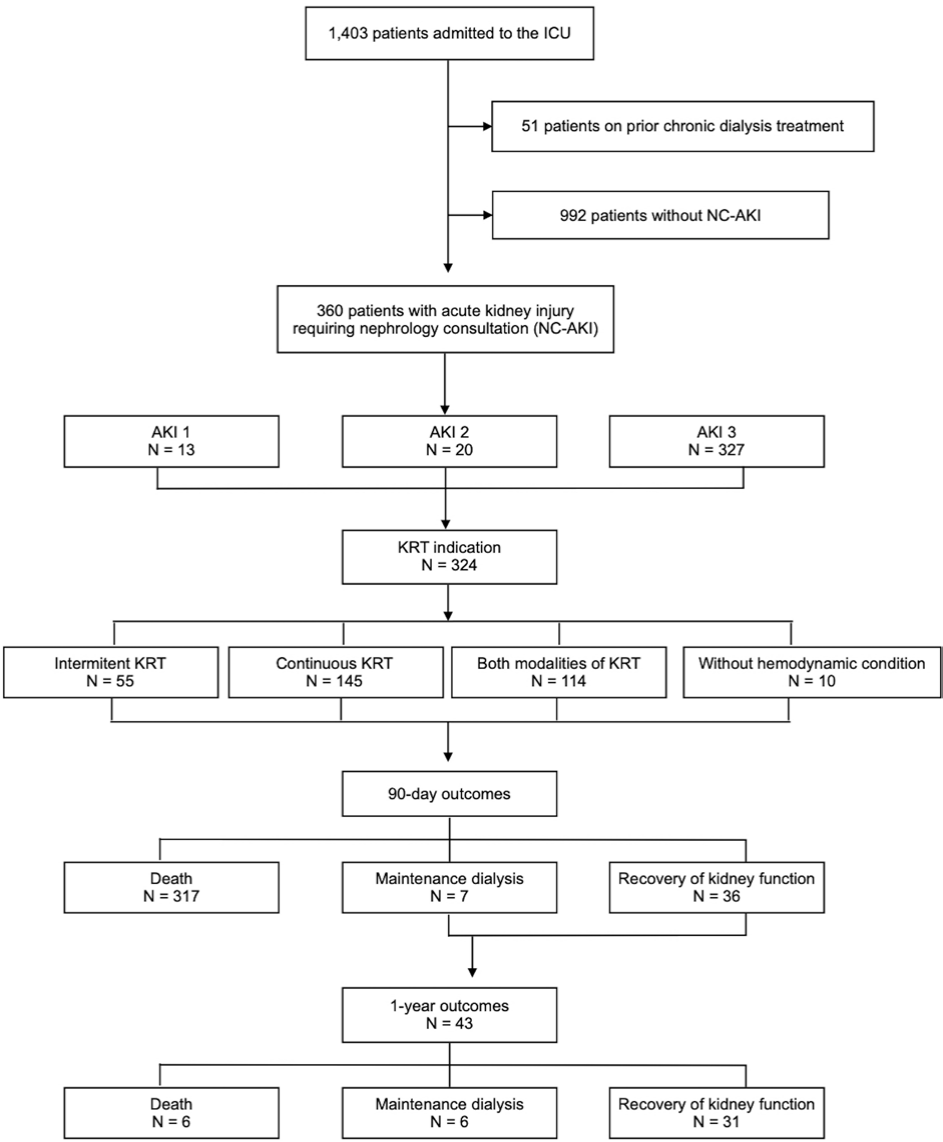
Flowchart of patients with COVID-19 hospitalized in the ICU during the study period. Patients with acute kidney injury (AKI) requiring nephrology consultation (NC-AKI) were included in the analysis. Outcomes were collected at 90 days and 1 year.

In the study period, 1,403 adult patients with COVID-19 needed intensive care admission at our institution. Of those, 360 (25.6%) patients presented AKI and nephrology consultation was required, fulfilling the inclusion criteria (NC-AKI). Figure 1 shows the study flowchart. Table 1 presents the clinical and demographic characteristics: most patients (60.6%) were male, with a median age of 66.0 (57.0–72.0) years. Hypertension (68.6%), diabetes (38.1%), and obesity (39.4%) were the most frequent comorbidities. Median BMI was 29.0 (IQR 25.0–33.2) and mean baseline creatinine was 1.34 ± 0.98 mg/dL. Additionally, 102 (28.3%) patients had a documented smoking history.

**Table 1:** Characteristics of critically ill COVID-19 patients with acute kidney injury that required nephrology consultation in a tertiary hospital

	N = 360
**Baseline characteristics** Male gender (N,%) Age (years) (median, IQR) BMI (kg/m^2^) (median, IQR) Baseline creatinine (mg/dL) (mean, SD)	218 (60.6) 66.0 (57.0–72.0) 29.0 (25.0–33.2) 1.34 (0.98)
**Comorbidities (N,%)** Hypertension Diabetes mellitus Obesity Malignancy Organ transplant recipients Ischemic cardiopathy	247 (68.6) 137 (38.1) 142 (39.4) 64 (17.8) 59 (16.4) 61 (16.9)
**Hospitalization factors** Albuminuria (2+) AKI Stage (N,%) Stage 1 Stage 2 Stage 3 KRT (N,%) Yes No (without hemodynamic conditions) No indication Mechanical ventilation, (N,%)	106 (29.3) 13 (3.6) 20 (5.6) 327 (90.8) 314 (87.2) 10 (2.8) 36 (10.0) 352 (97.8)
**Laboratorial data (median, IQR)** CPK (U/L) D-dimer (mg/L) Ferritin (ng/mL) Fibrinogen (mg/dL) Procalcitonin (ng/mL) Lactate (mmol/L) C-reactive protein (mg/dL) Troponin (pg/mL)	219.0 (89.0–658.0) 4.4 (1.8–4.4) 1625 (848–2584) 529 (410–679) 1.3 (0.4–3.6) 3.7 (2.7–6.3) 266.4 (214.1–332.8) 45.9 (11.4–300.7)

Abbreviations - BMI, body mass index; AKI, acute kidney injury; KRT, kidney replacement therapy; CPK, creatine phosphokinase; CRP, C reactive protein.

During hospitalization, 352 (97.8%) patients required mechanical ventilation, 302 (84.1%) received anticoagulation protocol, 345 (96.1%) received steroids, and 351 (98.3%) presented superimposed bacterial infections.

AKI stages 1, 2, and 3 were detected in 13 (3.6%), 20 (5.6%), and 327 (90.8%) NC-AKI patients, respectively. There were no significant associations between baseline clinical comorbidities and AKI stages. Qualitative albuminuria (≥ 2+) on urine dipstick was detected in 106 (29.3%) patients.

KRT was indicated for 324 (90.0%) patients and performed in 314 (87.2%): 10 (2.8%) patients did not have hemodynamic conditions to undergo dialysis treatment. Intermittent KRT was performed in 55 (17.5%) patients for a median of 5 (2.0–9.0) days; continuous therapy in 145 (46.2%) patients for a median of 7 (3.0–11.0) days; and 114 (36.2%) patients received both therapies.

### 90-Day Survival

In the first 90 days after COVID-19 diagnosis, 317 (88.1%) patients died, all during hospitalization.

**Figure 2:**
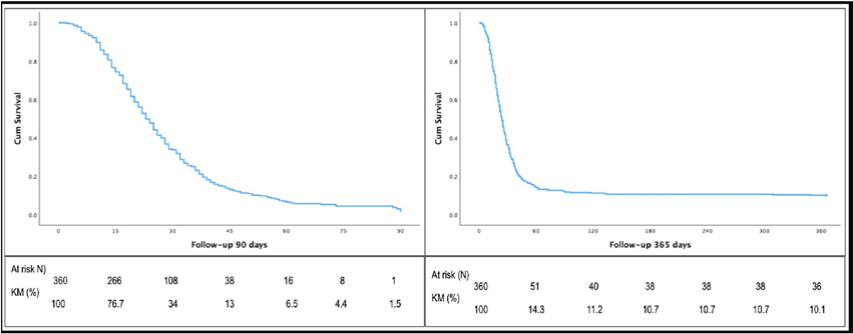
Left panel, 90-day survival of critically ill COVID-19 patients with acute kidney injury that required nephrology consultation. Right panel, 365-day survival of COVID-19 critically ill patients with acute kidney injury that required nephrology consultation.

Cumulative 90-day survival is presented in Figure 2. Female gender (p = 0.047), older age (p < 0.001), requirement of KRT (p < 0.0001), continuous KRT (p < 0.0001), AKI stage 3 (p = 0.005), malignancy (p = 0.048), mechanical ventilation (p < 0.0001), and superimposed bacterial infection (p < 0.0001) were significantly associated with death at 90 days (Table 2). The multivariate analysis for death at 90 days included variables with p < 0.20: age, gender, neoplasm, anticoagulation, mechanical ventilation, steroids, superimposed infection, dialysis, and C reactive protein. Table 3 shows the variables that remained associated with death at 90 days: older age [HR 1.015 (1.006–1.025), p = 0.002], female gender [HR 1.325 (1.059–1.659), p = 0.014], and no hemodynamic conditions for dialysis [HR 3.017 (1.435–6.342), p = 0.004].

**Table 2:** Risk factors for death at 90-days follow-up of critically ill COVID-19 patients with acute kidney injury that required nephrology consultation in a tertiary hospital

	Survival (N = 43)	Death (N = 317)	P value
Male gender (%)	32 (74.4)	186 (58.7)	**0.047**
Age (mean, DP)	57.7 (13.2)	64.8 (12.1)	**0.001**
BMI (Median, IQR)	30.4 (34.3–25.4)	28.7 (32.8–25.0)	0.466
AKI stage (N,%) Stage 1 Stage 2 Stage 3	5 (11.6) 4 (9.3) 34 (79.1)	8 (2.5) 16 (5.0) 293 (92.4)	**0.005**
KRT (N,%) Yes No condition No indication	31 (72.1) 0 12 (27.9)	283 (89.3) 10 (3.2) 24 (7.6)	**<0.001**
KRT modality (N,%) Intermittent Continuous Both	15 (34.8) 2 (4.7) 14 (32.6)	40 (12.6) 143 (45.1) 100 (31.5)	**<0.001**
Obesity (N,%)	23 (53.5)	119 (42.8)	0.189
Hypertension (N,%)	28 (65.1)	219 (69.1)	0.603
Diabetes mellitus (N,%)	15 (34.9)	122 (38.5)	0.739
Malignancy (N,%)	3 (7.0)	61 (19.2)	**0.048**
Ischemic cardiopathy (N,%)	6 (14.6)	55 (17.4)	0.657
Organ tranplant (N,%)	9 (20.9)	50 (36.3)	0.391
Mechanical ventilation (N,%)	37 (86.0)	314 (99.4)	**<0.001**
Anticoagulation	32 (74.4)	270 (85.4)	0.063
Superimposed infection	38 (88.4)	313 (99.7)	**<0.001**
Albuminuria (N,%)	15 (34.9)	91 (28.7)	0.347
CPK (U/L) (median, IQR)	141 (80–334)	227 (95–660)	0.160
D-dimer (mg/L) (median, IQR)	4.4 (2.1–4.4)	4.4 (1.7–4.4)	0.404
Ferritin (ng/mL) (mean, SD)	1643 (1746.7)	3350 (7256.7)	**0.023**
Fibrinogen (mg/dL) (mean, SD)	456.7 (244.7)	553.7 (192.6)	**0.035**
Lactate (mmol/dL) (mean, SD)	3 (1.1)	5.6 (4.1)	**<0.001**
Procalcitonin (ng/mL) (median, IQR)	0.8 (0.3–2.0)	1.4 (0.4–4.1)	0.119
CRP (mg/L) (median, IQR)	269.5 (203.8–311.8)	266.2 (217.3–335.1)	0.312
Troponin (pg/mL) (median, IQR)	29.1 (1.4–143.0)	54.3 (11.7–350.0)	0.179

Abbreviations - BMI, body mass index; AKI, acute kidney injury; KRT, kidney replacement therapy; CPK, creatine phosphokinase; CRP, C reactive protein.

**Table 3:** Variables associated with death at 90-day follow-up of critically ill COVID-19 patients with acute kidney injury requiring nephrology consultation

Death 90 days	Univariate			Multivariate		
	p	HR	CI 95%	p	HR	CI 95%
Age	**0.006**	1.014	1.004–1.024	**0.002**	1.015	1.006–1.025
Female gender	**0.028**	1.290	1.028–1.619	**0.014**	1.325	1.059–1.659
Dialysis No condition No indication	**0.004**	2.962 1	1.406–6.239	**0.004**	3.017 1	1.435–6.342

Note – Variables included in the model with no significant association on multivariate analysis: neoplasm, anticoagulation, mechanical ventilation, superimposed infection, steroid use, and C reactive protein.

**Figure 3:**
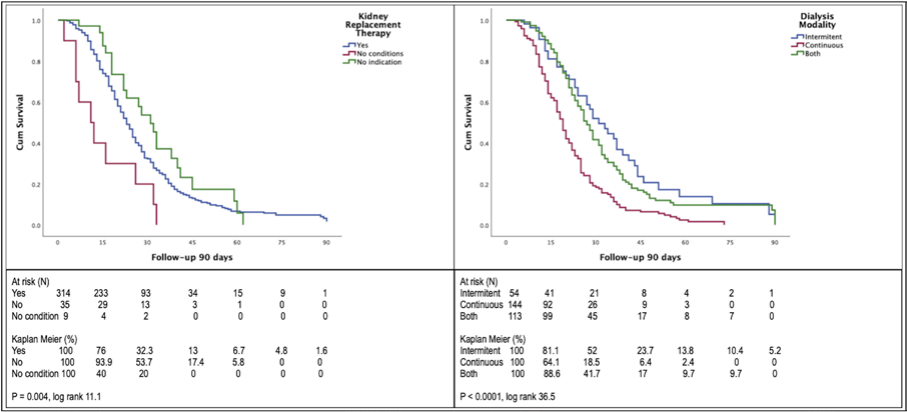
Left panel, 90-day survival of critically ill COVID-19 patients with acute kidney injury stratified by kidney replacement therapy requirement. Right panel, 90-day survival of critically ill COVID-19 patients with acute kidney injury that required kidney replacement therapy stratified by dialysis modality.

Figure 3 displays the 90-day survival, comparing patients that underwent KRT with those that did not require dialysis and those that did not have hemodynamic conditions to initiate therapy (median survival 23 vs. 31 vs. 11 days, respectively (p = 0.004, log rank 11.1). Of the 314 patients that underwent KRT, the 90-day median survival was lower in those that required continuous therapy compared to those that underwent intermittent dialysis only or that received both modalities (19 vs. 31 vs. 27 days, respectively (p < 0.0001, log rank 36.5).

### 90-Day Renal Outcomes

**Figure 4:**
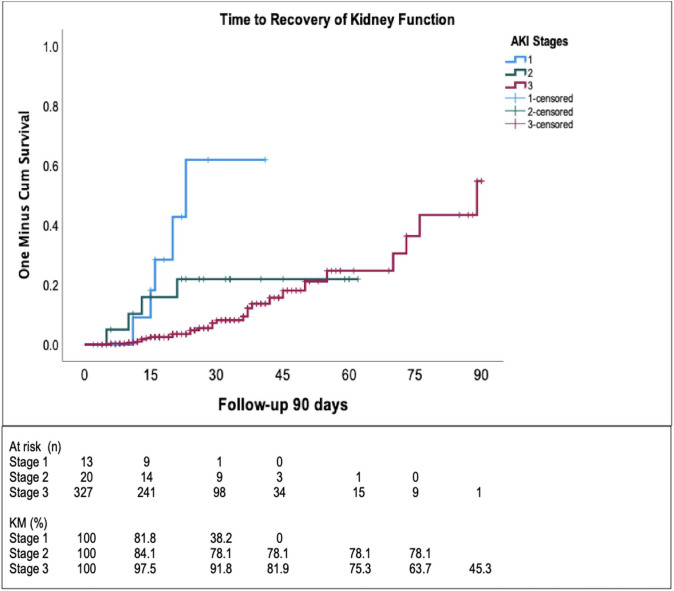
Time to recovery of kidney function (RKF) according to acute kidney injury (AKI) stages in patients that required nephrology consultation.

Forty-three (11.9%) patients survived beyond 90 days. At discharge, 7 (1.9%) remained on KRT and 36 (10%) had recovery of kidney function (RKF). There was an association of RKF at 90 days with no dialysis requirement (p = 0.024), anticoagulation (p = 0.036), and steroids use (p = 0.022). On multivariate analysis, only no KRT requirement remained significantly associated with RKF (HR 2.14, 95%CI 1.44–3.17, p = 0.009). Time to recovery of kidney function according to AKI stages is presented in Figure 4.

Notes – Stage 1: 5/13 (38.2%) patients had RKF in a median follow-up of 16.0 (13.0-21.5) days. Stage 2: 4/20 (20%) patients had RKF in a median follow-up of 11.5 (6.2-19.0) days. Stage 3: 27/327 (8.25%) patients had RKF in a median follow-up of 29.0 (15.0-45.0) days (log rank 30.6, p < 0.001).

KRT requirement, KRT dependence, and death were grouped as major adverse kidney events (MAKE) for composite analysis. A multivariate analysis with age, gender, diabetes, hypertension, obesity, neoplasm, ischemic cardiopathy, steroids, anticoagulation, and baseline creatinine yielded a significant association with female gender (HR 1.256, 95%CI 1.01–1.56, p = 0.041), older age (HR 1.01, 95%CI 1.003–1.022, p = 0.009), and higher baseline creatinine (HR 1.14, 95%CI 1.03–1.27, p = 0.009).

### One-Year Follow-up

The 43 patients that survived beyond 90 days were followed-up for 1 year: 6 (14%) died, 6 (14%) were receiving KRT (2 initiated and 4 remained), and 31 (72%) were free from dialysis. There were no significant associations of clinical variables with 1-year death.

Six (14%) patients that recovered kidney function had missing values of creatinine at one year. In the remaining 25 (58.1%) patients, the mean eGFR did not differ between baseline and discharge, but was significantly lower at 1-year follow-up (85.5 ± 23.6 vs. 81.7 ± 29.5 vs. 65.9 ± 24.8, p = 0.003) (Table 4).

**Table 4:** Comparison of eGFR at baseline, hospital discharge, and one year after COVID-19 diagnosis in critically ill patients with acute kidney injury requiring nephrology consultation

eGFR	Mean, SD	P value
Baseline	85.5 ± 23.6^a^	0.003
Hospital discharge	81.7 ± 29.5^a^	
One-year follow-up	65.9 ± 24.8^b^	

Abbreviations - eGFR, estimated glomerular filtration rate (CKD-EPI). Post-hoc Sidak test (p > 0.05).

## Discussion

In this prospective single-center cohort study we described critically ill COVID-19 patients with AKI that required nephrologist consultation (NC-AKI) in the ICUs of a tertiary hospital, representing 25% of ICU admissions. Most patients were male, in their sixth decade of life, with hypertension. The majority of patients presented stage 3 AKI and required kidney replacement therapy (KRT). Mortality within 90 days of follow-up was high. Amongst the surviving patients, most had recovery of kidney function at one-year follow-up, although with lower eGFR compared to baseline.

Our NC-AKI frequency was similar to other studies (22–36%)^1,4,14,15^ and lower than other Brazilian reports (55%)^20,21^. The reported incidence of AKI depends on the severity of disease, the need for hospitalization, the criteria adopted to define AKI, and the study design, and has changed over time as more data on the extra-pulmonary implications of this new disease have become available^22^.

The pathogenic mechanism by which SARS-CoV-2 affects the kidney is likely multifactorial, involving both viral effects^5^ and known risk factors for AKI^6^. The most frequent kidney biopsy finding in COVID-19 patients is acute tubular injury^11,12^, reflecting the numerous indirect causes of AKI seen in critical illness. These include hemodynamic instability, acute respiratory distress syndrome, exposure to nephrotoxins, hypoxia, cytokine storm, rhabdomyolysis, and secondary infections^5,13^. Additionally, detection of SARS-CoV-2 RNA^7,8,9^ and live virus^7^ in the kidney supports the hypothesis that the virus may directly target the kidneys via angiotensin-converting enzyme-2 receptors found in the proximal tubule cells and podocytes^10^. Its cytopathic effects can induce renal tubular injury during infection and replication, and the immune response can promote fibrosis and apoptosis^5^.

Our patients demographic data (gender, age, and comorbidities) were similar to other Brazilian reports^20,21^, aligned with the main risk factors associated with disease severity^15,23,24^. We focused on ICU patients that required nephrological consultation during a surge that required 3-fold augmentation of the hospital’s capacity to receive referrals from lower resource settings. Nearly all patients were on mechanical ventilation, received steroids and anticoagulation drugs, and presented superimposed bacterial infections. The use of nephrotoxic drugs was abundant. Hence, the elevated incidence of stage 3 AKI was not surprising, although higher than in previous studies (90% vs. 10–42%^1,14,15,25^).

The need for KRT was also higher than in most studies (90 vs. 10-33%^14,15,24,25^), probably explained by the baseline comorbidities, AKI, and disease severity. The mean baseline creatinine of 1.34 mg/dL may reflect a higher prevalence of CKD on the study population.

We found that older age, AKI stage 3, KRT requirement, continuous KRT, and mechanical ventilation were all associated with risk of death at 90 days, consistent with the patients’ severe clinical conditions and with other reports^14,15,21,25^. The COVID-19-associated AKI-KRT mortality has been reported as higher compared to critically ill patients without COVID-19^26^. These differences in outcomes may reflect the severe multiorgan failure associated with COVID-19 during the initial surge before the advent of vaccines^17,25^. Moreover, many of the studies of AKI among patients with COVID-19, including ours, have been conducted in academic hospitals, which tend to receive patients with more severe disease^15,16^. Our in-hospital mortality was 88%, which is high compared to other studies involving critically ill COVID-19 patients (33–50%)^2,20,21,24,25^.

Factors such as age, race, gender, obesity, diabetes, hypertension, cardiovascular disease, low eGFR, high IL-6 levels, mechanical ventilation, and vasoactive drugs have been identified as independent predictors of AKI^14,15^. Approximately 35–50%^1,14^ of patients hospitalized with COVID-19 and AKI do not fully recover their kidney function by hospital discharge. In our study, 12% of individuals were alive at 90 days after COVID-19 diagnosis, and 83% of these had RKF. The lack of a standardized definition for AKI recovery complicates comparison between studies. Some authors consider KFR as cessation of KRT, reporting rates of 41–92%^16,17,25,27^ among surviving patients. Ng et al.^28^ reported that 74% of their patients had RKF, defined as a decline of 33% from the peak admission serum creatinine level.

Although short-term outcomes of patients with AKI are widely reported^29,30,31,32^, data on long-term follow up of COVID-19 survivors with in-hospital AKI are more limited^4,18,33^. The presence of lung fibrosis observed after pulmonary infections from other coronavirus strains^34^ has raised concerns that COVID-19-associated AKI might induce tubulointerstitial fibrosis^35^, a potential pathway for progression from AKI to CKD^36,37^. The long-term decline in kidney function after AKI has been described in specific scenarios, such as patients discharged after ICU admission or those who underwent coronary angiography^38,39^. Understanding how COVID-19-related AKI impacts the long-term trajectory of kidney function can help clinicians tailor specific and thorough monitoring plans for managing kidney disease for recovering patients.

At one year follow up, 14% of survivors remained on KRT, another 14% died, and 72% were free from dialysis. Renal function analysis of this later subgroup showed that eGFR did not differ from baseline to hospital discharge. We have to consider the possibility of lower eGFR accuracy due to loss of muscular mass in the majority of patients. However, median eGFR one-year post-diagnosis was lower than both baseline and discharge levels, indicating partial recovery of kidney function.

The strengths of our study include its prospective nature and the assessment of patient survival and renal outcomes up to one year after COVID-19 infection. The limitations include the single-center nature with a population that required nephrological consultation, therefore limiting the generalizability of findings. Also, we recognize that the lack of measurement of urinary output for AKI staging, the use of qualitative albuminuria, and the missing creatinine measurements prior to hospitalization to define baseline eGFR are shortfalls in our analysis. Furthermore, some patients with AKI stage 1 and stage 2 may have been omitted from nephrological consultation by a possible brief self-limiting kidney injury. Another relevant aspect is the lack of a control group of patients with severe COVID-19 who did not develop AKI.

In conclusion, critically ill COVID-19 patients with NC-AKI presented a high frequency of AKI stage 3 and KRT requirement, with a high 90-day mortality. Surviving patients had high rates of recovery of kidney function, with lower eGFR at one-year follow-up compared to baseline.
