# Supplementary material for: Acute kidney injury in critically ill COVID-19 patients in a tertiary hospital: short and long-term kidney and patient outcomes
Source: J Bras Nefrol. 2025 Jan 10;47(1):e20240107. doi: 10.1590/2175-8239-JBN-2024-0107en (PMC11726861; doi:10.1590/2175-8239-JBN-2024-0107en)
Supplement: Translation

# Translation: Injúria renal aguda em pacientes críticos com COVID-19 em um hospital terciário: desfechos renais e de pacientes a curto e longo prazo

**DOI:** 10.1590/2175-8239-JBN-2024-0107pt

**Authors:** Juliana Alves Manhães de Andrade, Gisele Meinerz, Raphael Palma, Eduardo Rech, Marco Antônio Vinciprova Dall’Agnese, Cristiane Bundchen, Fernanda Bordignon Nunes, Gisele Branchini, Elizete Keitel

**Affiliations:** 1 Santa Casa de Porto Alegre, Porto Alegre, RS, Brasil.; 2 Universidade Federal de Ciências da Saúde de Porto Alegre, Programa de Pós-Graduação em Patologia, Porto Alegre, RS, Brasil.; 3 Universidade Federal de Ciências da Saúde de Porto Alegre, Porto Alegre, RS, Brasil.

**Keywords:** COVID-19, Injúria Renal Aguda, Mortalidade

## Abstract

**Introdução:**

IRA no contexto da COVID-19 associa-se a piores desfechos clínicos e renais, com dados limitados de longo prazo.

**Objetivo:**

Avaliar pacientes críticos com COVID-19 e IRA com necessidade de consultoria nefrológica (IRA-CN) em hospital terciário.

**Métodos:**

Coorte prospectiva de centro único de pacientes adultos críticos com COVID-19 com IRA-CN de 01/05/2020 a 30/04/2021. Avaliou-se terapia renal substitutiva (TRS), recuperação da função renal e óbito em 90 dias e 1 ano.

**Resultados:**

Incluímos 360 pacientes, 60,6% homens, idade mediana de 66,0 (57,0-72,0) anos, 38,1% com diabetes, 68,6% com hipertensão. Detectamos estágios 1, 2 e 3 de IRA em 3,6%, 5,6% e 90,8% dos pacientes, respectivamente. TRS foi indicada em 90% dos pacientes. No acompanhamento de 90 dias, 88,1% dos indivíduos morreram e 10,0% haviam recuperado a função renal. Sexo feminino (p = 0,047), idade avançada (p = 0,047), IRA estágio 3 (p = 0,005), necessidade de TRS (p < 0,0001), ventilação mecânica (p < 0,0001) e infecção bacteriana sobreposta (p < 0,0001) foram significativamente associados a óbito em 90 dias. Em um ano, a mortalidade foi 89,3%. Entre os sobreviventes, 72% dos pacientes recuperaram função renal, embora com TFGe significativamente menor ao valor basal (85,5 ± 23,6 vs. 65,9 ± 24,8 mL/min; p = 0,003).

**Conclusão:**

Pacientes críticos com COVID-19 com IRA-CN apresentaram alta frequência de IRA estágio 3 e necessidade de TRS, com elevada mortalidade em 90 dias. Pacientes sobreviventes apresentaram altas taxas de recuperação da função renal, com TFGe menor em um ano de acompanhamento comparado ao valor basal.

## Introdução

A doença do coronavírus-19 (COVID-19) tornou-se uma emergência de saúde poucos meses após o primeiro caso ter sido descrito em Wuhan, China, em dezembro de 2019. A injúria renal aguda (IRA) no contexto da infecção por COVID-19 está associada a piores desfechos clínicos e renais^1,2,3,4^. Os possíveis mecanismos para lesão renal incluem efeitos virais diretos e indiretos^5^, interagindo com fatores de risco comuns e conhecidos para IRA^6^.

A demonstração de RNA viral^7,8,9^ e vírus vivo^7^ em biópsias renais corrobora a hipótese de haver tropismo direto do SARS-CoV-2 para as células do túbulo proximal e podócitos, possivelmente via receptor da enzima conversora de angiotensina-2 expresso nessas células^10^. O SARS-CoV-2 é um vírus citopático que pode induzir diretamente lesão tubular renal durante a infecção e replicação, e a resposta imune pode promover fibrose e apoptose^5^. Necrose tubular aguda (NTA)^11,12^, microangiopatia trombótica e glomeruloesclerose segmentar e focal (GESF) colapsante são achados histopatológicos relatados^6^.

Os efeitos indiretos são aqueles associados a doenças graves, incluindo instabilidade hemodinâmica, hipovolemia, hipóxia, tempestade de citocinas, rabdomiólise, exposição a nefrotoxinas, disfunção endotelial, coagulopatia, infecções sobrepostas e *crosstalk* entre órgãos^5,13^. Os relatórios clínicos e de autópsia indicam que a infecção por SARS-CoV-2 induz um estado de hipercoagulação, aumentando o risco de formação de coágulos, o que leva a infarto pulmonar, esplênico e renal^6^.

A incidência relatada de IRA em pacientes com COVID-19, especialmente aqueles que estão hospitalizados, varia dependendo da gravidade da doença no grupo de pacientes analisados. Em dois grandes estudos observacionais com mais de 5.000 pacientes hospitalizados com COVID-19, a IRA foi relatada em 32 a 37% dos pacientes^14,15^. Aproximadamente metade dos pacientes com IRA não alcança a recuperação completa da função renal até a alta hospitalar^14^. Os preditores independentes de IRA incluem idade, raça, sexo, obesidade, diabetes, hipertensão, doenças cardiovasculares, baixa taxa de filtração glomerular estimada (TFGe) basal, níveis mais elevados de interleucina-6 (IL-6) ou necessidade de ventilação mecânica e drogas vasoativas^14,15^.

Existem dados limitados sobre a progressão da IRA e os desfechos de longo prazo em pacientes críticos com COVID-19. A maioria dos relatos avaliou desfechos hospitalares^13,14^ e desfechos de até 90 dias^16,17^ com um menor número de estudos apresentando acompanhamentos mais longos^4,18^. Como a IRA é um fator de risco clinicamente significativo para a doença renal crônica (DRC), as sequelas de longo prazo da IRA associada à COVID-19 merecem uma investigação mais aprofundada.

Nosso objetivo foi descrever o perfil clínico e laboratorial, bem como o curso clínico subsequente de uma amostra de pacientes críticos com COVID-19 e IRA que necessitaram de consultoria nefrológica em um hospital terciário. Os principais desfechos foram mortalidade em 90 dias e recuperação da função renal, além de sobrevida e taxa de filtração glomerular estimada em um ano.

## Métodos

### Desenho

Realizamos um estudo de coorte prospectivo com pacientes adultos internados nas Unidades de Terapia Intensiva (UTI) de um hospital terciário no sul do Brasil devido à infecção por COVID-19 entre 1º de maio de 2020 e 30 de abril de 2021.

### Objetivo

Nosso objetivo principal foi avaliar a sobrevida em curto (90 dias) e longo prazo (1 ano) de uma amostra de pacientes críticos com COVID-19 e IRA com necessidade de consultoria nefrológica (IRA-CN) em um hospital terciário. O objetivo secundário foi descrever o perfil clínico e laboratorial dos pacientes, os estágios da IRA e a necessidade de terapia renal substitutiva (TRS) durante a hospitalização. Por fim, foi avaliada a recuperação da função renal ou a necessidade de diálise após a alta hospitalar e no acompanhamento de um ano.

### Pacientes

Pacientes adultos (≥ 18 anos de idade) com COVID-19 internados na UTI com IRA-CN. Foram excluídos os pacientes com doença renal em estágio terminal em terapia renal substitutiva (TRS) crônica.

### Ética

O estudo foi aprovado pelo comitê de ética local (número de protocolo: 4237991). Um termo de consentimento informado foi obtido dos pacientes ou de seus parentes mais próximos.

### Coleta de Dados

Os dados clínicos e laboratoriais foram extraídos dos prontuários médicos eletrônicos (PME). Os dados demográficos incluíram idade, sexo e índice de massa corporal (IMC). Foram registradas comorbidades como hipertensão (HAS), obesidade, diabetes mellitus (DM), malignidade e doença arterial coronariana (DAC). Os exames laboratoriais realizados na admissão e durante o acompanhamento incluíram creatinina sérica, proteína C-reativa, D-dímero, ferritina, troponina-T, fibrinogênio, lactato e urinálise.

### Definições

A infecção por COVID-19 foi confirmada por meio da reação em cadeia da polimerase quantitativa em tempo real (RT-PCR). O nível basal de creatinina foi definido como o valor médio de creatinina entre 7 e 365 dias antes da hospitalização, obtido do PME do hospital ou por meio da família do paciente. Na ausência dessa informação, o valor mínimo de creatinina durante a hospitalização foi utilizado como a creatinina basal. Os estágios de IRA foram definidos de acordo com os critérios do KDIGO (*Kidney Disease: Improving Global Outcomes*) da seguinte forma: estágio 1 como um aumento na creatinina sérica de 1,5 a 1,9 vezes o valor basal ou um aumento na creatinina sérica de 0,3 mg/dL em 48 horas; estágio 2 como um aumento de 2 a 2,9 vezes o valor basal; e estágio 3 como um aumento de pelo menos 3 vezes o valor basal. O débito urinário não foi utilizado para a definição de IRA devido à escassa documentação dessa variável. A albuminúria positiva foi definida como 2+ (ou 1 g/dL) na urinálise, uma vez que valores mais baixos podem ser devidos tanto à NTA quanto à nefrite intersticial aguda (NIA). A frequência e a modalidade de TRS - intermitente, contínua ou ambas - foram registradas. Não foi realizada diálise peritoneal. A recuperação da função renal foi definida como a independência da diálise ou creatinina sérica dentro de 50% do valor basal, de acordo com a *Acute Dialysis Quality Initiative* ^19^.

### Desfechos e Acompanhamento

 Os desfechos primários dentro de 90 dias após a infecção por COVID-19 incluíram óbito, recuperação da função renal e comprometimento da função renal com necessidade de diálise de manutenção. Os pacientes sobreviventes foram submetidos à análise de creatinina sérica na alta e aos 12 meses. Eles foram contatados por telefone e questionados sobre seu estado clínico. Aqueles que apresentaram valores faltantes de creatinina sérica foram convidados a coletar novas amostras de sangue. As taxas de filtração glomerular foram calculadas utilizando a equação CKD-EPI (*Chronic Kidney Disease Epidemiology Collaboration*) no início do estudo, na alta hospitalar e aos 12 meses.

### Análise Estatística

As variáveis contínuas são apresentadas como mediana com intervalo interquartil (IIQ) ou média e desvio padrão (DP) de acordo com sua distribuição. A suposição de normalidade foi verificada pelo teste de Shapiro-Wilk. As variáveis categóricas são expressas como valores absolutos acompanhados de porcentagens. Para a associação entre dados demográficos, características clínicas e achados laboratoriais, e óbito em 90 dias e 1 ano foram utilizados o teste χ^2^, o teste exato de Fisher e o teste U de Mann-Whitney, conforme a natureza e a distribuição das variáveis. As variáveis com P < 0,20 foram incluídas nas análises multivariadas. As curvas de sobrevida foram construídas com Kaplan-Meier e comparadas pelo teste de log-rank. A análise de regressão de riscos proporcionais de Cox foi utilizada para determinar as razões de risco nos acompanhamentos. Os valores de creatinina e a TFGe no início do estudo, na alta e um ano após a infecção por COVID-19 foram comparados por meio de ANOVA para variáveis repetidas e Sidak para comparações múltiplas. O IBM SPSS^®^ v. 25 foi utilizado para a análise dos dados. Os testes estatísticos foram bicaudais e os níveis de significância foram de 0,05.

## Resultados

No período do estudo, 1.403 pacientes adultos com COVID-19 necessitaram de internação em terapia intensiva em nossa instituição. Desses, 360 (25,6%) apresentaram IRA e foi necessária consultoria nefrológica, preenchendo os critérios de inclusão (IRA-CN). A Figura 1 mostra o fluxograma do estudo. A Tabela 1 apresenta as características clínicas e demográficas: a maioria dos pacientes (60,6%) era do sexo masculino, com mediana de idade de 66,0 (57,0–72,0) anos. Hipertensão (68,6%), diabetes (38,1%) e obesidade (39,4%) foram as comorbidades mais frequentes. O IMC mediano foi de 29,0 (IIQ 25,0–33,2) e a creatinina basal média foi de 1,34 ± 0,98 mg/dL. Além disso, 102 (28,3%) pacientes tinham histórico documentado de tabagismo.

**Tabela 1:** Características dos pacientes críticos com COVID-19 e injúria renal aguda que necessitaram de consultoria nefrológica em um hospital terciário

	N = 360
**Características basais** Sexo masculino (N,%) Idade (anos) (mediana, IIQ) IMC (kg/m^2^) (mediana, IIQ) Creatinina basal (mg/dL) (média, DP)	218 (60,6) 66,0 (57,0–72,0) 29,0 (25,0–33,2) 1,34 (0,98)
**Comorbidades (N,%)** Hipertensão Diabetes mellitus Obesidade Malignidade Receptores de transplante de órgãos Cardiopatia isquêmica	247 (68,6) 137 (38,1) 142 (39,4) 64 (17,8) 59 (16,4) 61 (16,9)
**Fatores de internação** Albuminúria (2+) Estágio da IRA (N,%) Estágio 1 Estágio 2 Estágio 3 TRS (N,%) Sim Não (sem condições hemodinâmicas) Sem indicação Ventilação mecânica, (N,%)	106 (29,3) 13 (3,6) 20 (5,6) 327 (90,8) 314 (87,2) 10 (2,8) 36 (10,0) 352 (97,8)
**Dados laboratoriais (mediana, IIQ)** CPK (U/L) D-dímero (mg/L) Ferritina (ng/mL) Fibrinogênio (mg/dL) Procalcitonina (ng/mL) Lactato (mmol/L) Proteína C-reativa (mg/dL) Troponina (pg/mL)	219,0 (89,0–658,0) 4,4 (1,8–4,4) 1625 (848–2584) 529 (410–679) 1,3 (0,4–3,6) 3,7 (2,7–6,3) 266,4 (214,1–332,8) 45,9 (11,4–300,7)

Abreviaturas - IMC, índice de massa corporal; IRA, injúria renal aguda; TRS, terapia renal substitutiva; CPK, creatina fosfoquinase; PCR, Proteína C reativa.

**Figura 1:** Fluxograma de pacientes com COVID-19 hospitalizados na UTI durante o período do estudo. Pacientes com injúria renal aguda (IRA) com necessidade de consultoria nefrológica (IRA-CN) foram incluídos na análise. Os desfechos foram coletados aos 90 dias e 1 ano.

Durante a hospitalização, 352 (97,8%) pacientes necessitaram de ventilação mecânica, 302 (84,1%) receberam protocolo de anticoagulação, 345 (96,1%) receberam esteroides e 351 (98,3%) apresentaram infecções bacterianas sobrepostas.

Os estágios 1, 2 e 3 da IRA foram detectados em 13 (3,6%), 20 (5,6%) e 327 (90,8%) pacientes com IRA-CN, respectivamente. Não houve associações significativas entre as comorbidades clínicas basais e os estágios de IRA. A albuminúria qualitativa (≥ 2+) no teste de urina com fita reagente foi detectada em 106 (29,3%) pacientes.

A TRS foi indicada para 324 (90,0%) indivíduos e realizada em 314 (87,2%): 10 (2,8%) pacientes não apresentaram condições hemodinâmicas para realizar o tratamento dialítico. A TRS intermitente foi realizada em 55 (17,5%) pacientes por uma mediana de 5 (2,0–9,0) dias; a terapia contínua em 145 (46,2%) pacientes por uma mediana de 7 (3,0–11,0) dias; e 114 (36,2%) pacientes receberam ambas as terapias.

### Sobrevida em 90 Dias

Nos primeiros 90 dias após o diagnóstico de COVID-19, 317 (88,1%) pacientes foram a óbito, todos durante a hospitalização.

A sobrevida cumulativa em 90 dias é apresentada na Figura 2. Sexo feminino (p = 0,047), idade mais avançada (p < 0,001), necessidade de TRS (p < 0,0001), TRS contínua (p < 0,0001), IRA estágio 3 (p = 0,005), malignidade (p = 0,048), ventilação mecânica (p < 0,0001) e infecção bacteriana sobreposta (p < 0,0001) foram significativamente associados a óbito em 90 dias (Tabela 2). A análise multivariada para óbito em 90 dias incluiu variáveis com p < 0,20: idade, sexo, neoplasia, anticoagulação, ventilação mecânica, esteroides, infecção sobreposta, diálise e proteína C reativa. A Tabela 3 mostra as variáveis que permaneceram associadas a óbito em 90 dias: idade avançada [HR 1,015 (1,006–1,025; p = 0,002)], sexo feminino [HR 1,325 (1,059–1,659); p = 0,014] e não ter condições de diálise [HR 3,017 (1,435–6,342); p = 0,004].

**Figura 2:** Painel esquerdo, sobrevida em 90 dias de pacientes críticos com COVID-19 com injúria renal aguda que necessitaram de consultoria nefrológica. Painel direito, sobrevida em 365 dias de pacientes críticos com COVID-19 com injúria renal aguda que necessitaram de consultoria nefrológica.

**Tabela 2:** Fatores de risco para óbito no acompanhamento de 90 dias de pacientes críticos com COVID-19 com injúria renal aguda que necessitaram de consultoria nefrológica em um hospital terciário

	Sobrevivente (N = 43)	Óbito (N = 317)	Valor de P
Sexo masculino (%)	32 (74,4)	186 (58,7)	**0,047**
Idade (média, DP)	57,7 (13,2)	64,8 (12,1)	**0,001**
IMC (mediana, IIQ)	30,4 (34,3–25,4)	28,7 (32,8–25,0)	0,466
Estágio da IRA (N,%) Estágio 1 Estágio 2 Estágio 3	5 (11,6) 4 (9,3) 34 (79,1)	8 (2,5) 16 (5,0) 293 (92,4)	**0,005**
TRS (N,%) Sim Sem condições Sem indicação	31 (72,1) 0 12 (27,9)	283 (89,3) 10 (3,2) 24 (7,6)	**<0,001**
Modalidade da TRS (N,%) Intermitente Contínua Ambas	15 (34,8) 2 (4,7) 14 (32,6)	40 (12,6) 143 (45,1) 100 (31,5)	**<0,001**
Obesidade (N,%)	23 (53,5)	119 (42,8)	0,189
Hipertensão (N,%)	28 (65,1)	219 (69,1)	0,603
Diabetes mellitus (N,%)	15 (34,9)	122 (38,5)	0,739
Malignidade (N,%)	3 (7,0)	61 (19,2)	**0,048**
Cardiopatia isquêmica (N,%)	6 (14,6)	55 (17,4)	0,657
Transplante de órgãos (N,%)	9 (20,9)	50 (36,3)	0,391
Ventilação mecânica (N,%)	37 (86,0)	314 (99,4)	**<0,001**
Anticoagulação	32 (74,4)	270 (85,4)	0,063
Infecção sobreposta	38 (88,4)	313 (99,7)	**<0,001**
Albuminúria (N,%)	15 (34,9)	91 (28,7)	0,347
CPK (U/L) (mediana, IIQ)	141 (80–334)	227 (95–660)	0,160
D-dímero (mg/L) (mediana, IIQ)	4,4 (2,1–4,4)	4,4 (1,7–4,4)	0,404
Ferritina (ng/mL) (média, DP)	1643 (1746,7)	3350 (7256,7)	**0,023**
Fibrinogênio (mg/dL) (média, DP)	456,7 (244,7)	553,7 (192,6)	**0,035**
Lactato (mmol/dL) (média, DP)	3 (1,1)	5,6 (4,1)	**<0,001**
Procalcitonina (ng/mL) (mediana, IIQ)	0,8 (0,3–2,0)	1,4 (0,4–4,1)	0,119
PCR (mg/L) (mediana, IIQ)	269,5 (203,8–311,8)	266,2 (217,3–335,1)	0,312
Troponina (pg/mL) (mediana, IIQ)	29,1 (1,4–143,0)	54,3 (11,7–350,0)	0,179

Abreviaturas - IMC, índice de massa corporal; IRA, injúria renal aguda; TRS, terapia renal substitutiva; CPK, creatina fosfoquinase; PCR, proteína C reativa.

**Tabela 3:** Variáveis associadas ao óbito no acompanhamento de 90 dias de pacientes críticos com COVID-19 e injúria renal aguda com necessidade de consultoria nefrológica

Óbito em 90 dias	Univariada			Multivariada		
	p	HR	IC 95%	p	HR	IC 95%
Idade	**0,006**	1,014	1,004–1,024	**0,002**	1,015	1,006–1,025
Sexo feminino	**0,028**	1,290	1,028–1,619	**0,014**	1,325	1,059–1,659
Diálise Sem condições Sem indicação	**0,004**	2,962 1	1,406–6,239	**0,004**	3,017 1	1,435–6,342

Nota – Variáveis incluídas no modelo sem associação significativa na análise multivariada: neoplasia, anticoagulação, ventilação mecânica, infecção sobreposta, uso de esteroides e proteína C reativa.

A Figura 3 mostra a sobrevida em 90 dias, comparando pacientes submetidos à TRS com aqueles que não necessitaram de diálise e aqueles que não apresentaram condições hemodinâmicas para iniciar a terapia (sobrevida mediana de 23 *vs.* 31 *vs.* 11 dias, respectivamente (p = 0,004; log rank 11,1). Dos 314 pacientes submetidos à TRS, a sobrevida mediana em 90 dias foi menor entre aqueles que necessitaram de terapia contínua em comparação aos que foram submetidos somente à diálise intermitente ou que receberam ambas as modalidades (19 *vs.* 31 *vs.* 27 dias, respectivamente (p < 0,0001; log rank 36,5).

**Figura 3:** Painel esquerdo, sobrevida em 90 dias de pacientes críticos com COVID-19 com injúria renal aguda estratificada por necessidade de terapia renal substitutiva. Painel direito, sobrevida em 90 dias de pacientes críticos com COVID-19 com injúria renal aguda que necessitaram de terapia renal substitutiva estratificada por modalidade de diálise.

### Desfechos Renais em 90 Dias

Quarenta e três (11,9%) pacientes sobreviveram além de 90 dias. Na alta, 7 (1,9%) permaneceram em TRS e 36 (10%) apresentaram recuperação da função renal (RFR). Houve uma associação da RFR em 90 dias com a não necessidade de diálise (p = 0,024), anticoagulação (p = 0,036) e uso de esteroides (p = 0,022). Na análise multivariada, apenas a ausência de necessidade de TRS permaneceu significativamente associada à RFR (HR 2,14; IC95% 1,44–3,17; p = 0,009). O tempo até a recuperação da função renal de acordo com os estágios da IRA é apresentado na Figura 4.

**Figura 4:** Tempo para a recuperação da função renal (RFR) de acordo com os estágios da injúria renal aguda (IRA) em pacientes que necessitaram de consultoria nefrológica.

Notas – Estágio 1: 5/13 (38,2%) pacientes apresentaram RFR em um acompanhamento mediano de 16,0 (13,0–21,5) dias. Estágio 2: 4/20 (20%) pacientes apresentaram RFR em um acompanhamento mediano de 11,5 (6,2–19,0) dias. Estágio 3: 27/327 (8,25%) pacientes apresentaram RFR em um acompanhamento mediano de 29,0 (15,0–45,0) dias (log rank 30,6; p < 0,001).

Necessidade de TRS, dependência de TRS e óbito foram agrupados como eventos renais adversos maiores (MAKE, por sua sigla em inglês) para análise composta. Uma análise multivariada com idade, sexo, diabetes, hipertensão, obesidade, neoplasia, cardiopatia isquêmica, esteroides, anticoagulação e creatinina basal gerou uma associação significativa com sexo feminino (HR 1,256; IC95% 1,01–1,56; p = 0,041), idade mais avançada (HR 1,01; IC95% 1,003–1,022; p = 0,009) e creatinina basal mais elevada (HR 1,14; IC95% 1,03–1,27; p = 0,009).

### Acompanhamento de um ano

Os 43 pacientes que sobreviveram além de 90 dias foram acompanhados por um ano: 6 (14%) foram a óbito, 6 (14%) estavam recebendo TRS (2 iniciaram e 4 permaneceram) e 31 (72%) estavam livres do tratamento dialítico. Não houve associações significativas de variáveis clínicas com o óbito em um ano.

Seis (14%) pacientes que recuperaram a função renal não apresentaram valores de creatinina em um ano. Nos 25 (58,1%) pacientes restantes, a TFGe média não diferiu entre o valor basal e a alta hospitalar, mas foi significativamente menor em um ano de acompanhamento (85,5 ± 23,6 *vs.* 81,7 ± 29,5 *vs.* 65,9 ± 24,8; p = 0,003) (Tabela 4).

**Tabela 4:** Comparação da TFGe no início do estudo, na alta hospitalar e um ano após o diagnóstico de COVID-19 em pacientes críticos com injúria renal aguda

TFGe	Média, DP	Valor de P
Basal	85,5 ± 23,6^a^	0,003
Alta hospitalar	81,7 ± 29,5^a^	
Acompanhamento de um ano	65,9 ± 24,8^b^	

Abreviaturas - TFGe, taxa de filtração glomerular estimada (CKD-EPI). Teste post-hoc de Sidak (p > 0,05).

## Discussão

Neste estudo de coorte prospectivo de centro único, descrevemos pacientes críticos com COVID-19 e IRA com necessidade de consultoria nefrológica (IRA-CN) nas UTI de um hospital terciário, representando 25% das admissões em UTI. A maioria dos pacientes era do sexo masculino, em sua sexta década de vida e apresentava hipertensão. A maioria dos pacientes apresentou IRA estágio 3 e necessitou de terapia renal substitutiva (TRS). A mortalidade dentro de 90 dias de acompanhamento foi elevada. Entre os pacientes sobreviventes, a maioria apresentou recuperação da função renal no acompanhamento de um ano, embora com TFGe mais baixa em comparação ao valor basal.

Nossa frequência de IRA-CN foi semelhante à de outros estudos (22-36%^1,4,14,15^) e inferior à de outros relatos brasileiros (55%)^20,21^. A incidência relatada de IRA depende da gravidade da doença, da necessidade de hospitalização, dos critérios adotados para definir a IRA e do desenho do estudo. Essa incidência mudou ao longo do tempo, à medida que mais dados sobre as implicações extrapulmonares dessa nova doença se tornaram disponíveis^22^.

O mecanismo patogênico pelo qual o SARS-CoV-2 afeta o rim é provavelmente multifatorial, envolvendo tanto efeitos virais^5^ quanto fatores de risco conhecidos para IRA^6^. O achado mais frequente de biópsia renal em pacientes com COVID-19 é a lesão tubular aguda^11,12^, refletindo as inúmeras causas indiretas de IRA observadas em doenças críticas. Essas incluem instabilidade hemodinâmica, síndrome da angústia respiratória aguda, exposição a nefrotoxinas, hipóxia, tempestade de citocinas, rabdomiólise e infecções secundárias^5,13^. Além disso, a detecção do RNA do SARS-CoV-2^7,8,9^ e do vírus vivo^7^ nos rins sustenta a hipótese de que o vírus pode atingir diretamente os rins por meio dos receptores da enzima conversora de angiotensina-2 encontrados nas células do túbulo proximal e nos podócitos^10^. Seus efeitos citopáticos podem induzir lesão tubular renal durante a infecção e a replicação, e a resposta imune pode promover fibrose e apoptose^5^.

Os dados demográficos dos nossos pacientes (sexo, idade e comorbidades) foram semelhantes a de outros relatos brasileiros^20,21^, alinhados com os principais fatores de risco associados à gravidade da doença^15,23,24^. Nosso foco foi em pacientes de UTI que necessitaram de consultoria nefrológica durante um surto de COVID’19 que demandou um aumento de três vezes na capacidade do hospital para receber encaminhamentos de locais com menos recursos. Quase todos os pacientes estavam em ventilação mecânica, receberam esteroides e anticoagulantes e apresentaram infecções bacterianas sobrepostas. O uso de medicamentos nefrotóxicos foi abundante. Portanto, a alta incidência de IRA estágio 3 não foi surpreendente, embora tenha sido maior do que em estudos anteriores (90% *vs.* 10–42%^1,14,15,25^).

A necessidade de TRS também foi superior à da maioria dos estudos (90 *vs.* 10–33%)^14,15,24,25^, provavelmente explicada pelas comorbidades basais, IRA e gravidade da doença. A creatinina basal média de 1,34 mg/dL pode refletir uma maior prevalência de DRC na população do estudo.

Constatamos que idade avançada, IRA estágio 3, necessidade de TRS, TRS contínua e ventilação mecânica foram todos associados ao risco de óbito em 90 dias, o que está de acordo com a gravidade das condições clínicas dos pacientes e com outros relatos^14,15,21,25^. A mortalidade por IRA-TRS associada à COVID-19 foi relatada como sendo mais elevada em comparação com pacientes críticos sem COVID-19^26^. Essas diferenças nos desfechos podem refletir a grave falência múltipla de órgãos associada à COVID-19 durante o surto inicial, antes do advento das vacinas^17,25^. Além disso, muitos dos estudos sobre IRA entre pacientes com COVID-19, incluindo o nosso, foram realizados em hospitais universitários, que tendem a receber pacientes com doença mais grave^15,16^. Nossa mortalidade hospitalar foi de 88%, um índice elevado em comparação com outros estudos envolvendo pacientes críticos com COVID-19 (33–50%)^2,20,21,24,25^.

Fatores como idade, raça, sexo, obesidade, diabetes, hipertensão, doença cardiovascular, baixa TFGe, níveis elevados de IL-6, ventilação mecânica e drogas vasoativas foram identificados como preditores independentes de IRA^14,15^. Aproximadamente 35–50%^1,14^ dos pacientes hospitalizados com COVID-19 e IRA não recuperam totalmente sua função renal até a alta hospitalar. Em nosso estudo, 12% dos indivíduos estavam vivos aos 90 dias após o diagnóstico de COVID-19, e 83% deles apresentaram RFR. A falta de uma definição padronizada para a recuperação da IRA dificulta a comparação entre estudos. Alguns autores consideram RFR como a interrupção da TRS, relatando taxas de 41–92%^16,17,25,27^ entre os pacientes sobreviventes. Ng et al.^28^ relataram que 74% de seus pacientes apresentaram RFR, definida como um declínio de 33% em relação ao nível máximo de creatinina sérica na admissão.

Embora os desfechos de curto prazo de pacientes com IRA sejam amplamente relatados^29,30,31,32^, os dados sobre o acompanhamento em longo prazo de sobreviventes da COVID-19 com IRA hospitalar são mais limitados^4,18,33^. A presença de fibrose pulmonar observada após infecções pulmonares causadas por outras cepas do coronavírus^34^ levantou preocupações de que a IRA associada à COVID-19 possa induzir fibrose tubulointersticial^35^, uma possível via de progressão de IRA para DRC^36,37^. O declínio em longo prazo da função renal após IRA foi descrito em cenários específicos, como pacientes que receberam alta após admissão em UTI ou aqueles que foram submetidos à angiografia coronariana^38,39^. Compreender como a IRA relacionada à COVID-19 impacta a trajetória a longo prazo da função renal pode auxiliar os médicos a adaptarem planos de monitoramento específicos e abrangentes para o manejo da doença renal em pacientes em recuperação.

No acompanhamento de um ano, 14% dos sobreviventes permaneceram em TRS, outros 14% foram a óbito e 72% estavam livres de diálise. A análise da função renal desse último subgrupo revelou que a TFGe não diferiu desde o início até a alta hospitalar. É preciso considerar a possibilidade de menor precisão da TFGe devido à perda de massa muscular na maioria dos pacientes. No entanto, a TFGe mediana um ano após o diagnóstico foi inferior aos níveis basais e da alta hospitalar, indicando recuperação parcial da função renal.

Os pontos fortes do nosso estudo incluem sua natureza prospectiva e a avaliação da sobrevida do paciente e dos desfechos renais até um ano após a infecção por COVID-19. As limitações incluem a natureza de centro único com uma população que necessitou de consultoria nefrológica, limitando, portanto, a generalização dos achados. Além disso, reconhecemos que a falta de medição do débito urinário para o estadiamento da IRA, o uso de albuminúria qualitativa e a ausência de medições de creatinina antes da hospitalização para definir a TFGe basal são deficiências em nossa análise. Ainda, alguns pacientes com IRA estágio 1 e estágio 2 podem ter sido omitidos da consultoria nefrológica por uma possível injúria renal breve e autolimitada. Outro aspecto relevante é a falta de um grupo controle de pacientes com COVID-19 grave que não desenvolveram IRA.

Em conclusão, os pacientes críticos com COVID-19 com IRA-CN apresentaram uma alta frequência de IRA estágio 3 e necessidade de TRS, com elevada mortalidade em 90 dias. Os pacientes sobreviventes apresentaram altas taxas de recuperação da função renal, com TFGe mais baixa em um ano de acompanhamento em comparação com o valor basal.
